# The Effect of Type-2 Diabetes Mellitus on Sleep Architecture and Sleep Apnea Severity in Patients With Obstructive Sleep Apnea Syndrome

**DOI:** 10.7759/cureus.61215

**Published:** 2024-05-28

**Authors:** Büşra Durak, Canan Gunduz Gurkan, Duygu Özol, Sema Saraç

**Affiliations:** 1 Department of Pulmonology, Hitit University Faculty of Medicine, Çorum, TUR; 2 Department of Pulmonology, Süreyyapaşa Chest Diseases and Thoracic Surgery Training and Research Hospital, Istanbul, TUR

**Keywords:** diabetes control, sleep quality, diabetes mellitus, sleep architecture, obstructive sleep apnea syndrome

## Abstract

Introduction: Obstructive sleep apnea syndrome (OSAS) is a severe condition that is characterized by recurrent partial or complete breathing interruptions during sleep, leading to insulin resistance, microvascular complications, and cardiovascular complications. It is of great importance to know the impact of type 2 diabetes mellitus (DM), which is prevalent in the world and in our country, Turkey, leads to significant mortality and morbidity, significantly affects the quality of life, and requires continuous follow-up, on sleep in patients with OSAS and to raise awareness on this issue. In this study, we aimed to determine the effects of diabetes on sleep duration and sleep architecture in patients with OSAS and to investigate the relationship between OSAS severity and DM control.

Methods: Fifty diabetic and 42 non-diabetic patients diagnosed with OSAS at the Sleep Disorders Center of Süreyyapaşa Chest Diseases and Thoracic Surgery Training and Research Hospital, Istanbul, Turkey, between October 2022 and March 2023 were included in the study. Polysomnographic and biochemical parameters of the two groups were compared. The effect of OSAS severity and sleep architecture on diabetes control was investigated.

Results: No significant difference was found between diabetic and non-diabetic patients in terms of total sleep duration, sleep efficiency, and sleep latency, whereas REM (rapid eye movement) latency was prolonged and REM sleep duration and percentage were significantly lower in diabetic patients. The severity of OSAS was found to be greater in diabetic patients and they spent significantly more time below 90% saturation during sleep. No correlation was found between the groups in the glycated hemoglobin (HbA1c) parameter, which we examined in terms of diabetes control, sleep architecture, and OSAS severity.

Conclusion: The presence of diabetes aggravates the severity of OSAS, prolongs the transition to REM sleep, and leads to a decrease in REM duration. Sleep is essential for both mental and physical well-being. In this regard, it is of utmost importance to examine diabetic patients for OSAS and to perform polysomnography in appropriate patients.

## Introduction

Obstructive sleep apnea syndrome (OSAS) is a common disease caused by recurrent partial (hypopnea) or complete (apnea) obstruction of the airway during sleep throughout the night, which seriously affects people's lives [1]. Increased respiratory effort as a result of recurrent apneas leads to arousals, leading to frequent sleep interruptions, increased sympathetic activity, hyperinflammation, and intermittent oxygen deprivation. With all these underlying mechanisms, OSAS progresses with serious cardiovascular and metabolic comorbidities [2-4]. Sleep is defined as a temporary, periodic state of unconsciousness, reversible by various stimuli, which ensures the conservation of energy, development, and repair of the body. Sleep architecture is considered a composition of REM (rapid eye movement) and non-REM periods. Each sleep cycle consists of 90-120 minutes of non-REM (stage 1-2-3) and REM phases. Approximately 1-5% of sleep is spent in stage 1, 50% in stage 2, 20-25% in stage 3, and 20-25% in the REM period [5]. In the first half of sleep, non-REM stage 3 is longer, while in the second half of the night, this period shortens, and REM duration increases. Sleep latency is the time from the beginning of recording until the first sleep phase (usually stage 1) is observed. It is generally expected to fall asleep in 15-20 minutes and the first REM latency to be observed in 90-120 minutes. REM sleep is known to be important for learning and memory consolidation [6]. Moreover, REM sleep facilitates cortical plasticity, which is defined as the brain's ability to undergo structural and physiological changes [7,8] and has been shown to increase overall creativity.

The number of people with type 2 diabetes mellitus (DM) worldwide was 463 million in 2019, but this number is expected to rise to 578 million by 2035, a 25% increase. With an increasing incidence and common complications, DM is a serious disease [9,10]. When sleep duration and quality are impaired, the risk of developing obesity, hyperglycemia, and insulin resistance increases due to changes in the release of hormones that provide energy balance, and micro and macrovascular complications occur in people with DM [11]. The prevalence of OSAS is estimated to be approximately 5%, while the prevalence of type 2 DM was found to be 6.4% in adults. Common pathophysiological mechanisms such as similar prevalence and obesity lead to the frequent occurrence of both diseases in the same individual [12,13]. In the Sleep AHEAD (Action for Health in Diabetes) study, which included 264 obese diabetic patients with a mean age of 61.2 years, the prevalence of concomitant OSAS was 86% [14], while in the Einhorn and Bloomgarden study, the prevalence of OSAS was 48% in 279 diabetic patients [15]. Meanwhile, Zhang et al. detected OSAS in 60% of 880 inpatients with type 2 DM using a two-channel portable sleep test [16]. The coexistence of these two diseases with similar risk factors has been shown in many studies. Hence, in our study, we aimed to determine the effects of diabetes on sleep duration and sleep architecture in patients with OSAS and to investigate the relationship between OSAS severity and DM control.

## Materials and methods

Patients hospitalized in the Sleep Disorders Center of Süreyyapaşa Chest Diseases and Thoracic Surgery Training and Research Hospital, Istanbul, Turkey, between October 2022 and March 2023 and diagnosed with OSAS following a polysomnography (PSG) examination and those who underwent glycated hemoglobin (HbA1C) test for any reason within one month were included. The patients were divided into two groups: diabetic and non-diabetic. Patients who had previously been diagnosed with diabetes and were currently undergoing treatment were included in the diabetic patient group when they presented to the clinic. Forty (80%) diabetic patients were using only oral anti-diabetic drugs, seven (14%) diabetic patients were using both oral anti-diabetic drugs and insulin, and three (6%) patients were using only insulin treatment. The non-diabetic group was selected based on HbA1c tests showing levels under 6%. Patients using alcohol, hypnosedatives, antidepressants, and antiepileptic drugs were excluded.

Medications, comorbidities, height and weight measurements taken on the same scale, complaints, HbA1c value, and PSG values were noted. PSG of all patients was performed with the Neurosoft PSG device (Neurosoft, OOO, Ivanovo, Russia) in the sleep laboratory under the supervision of a technician and during spontaneous sleep. Electroencephalography (EEG), electrooculography (EOG), mandibular and tibial electromyograms (EMGs), and electrocardiography (ECG) were recorded. Airflow was measured by a nasal-oral thermistor and respiratory effort by thoracoabdominal piezoelectric belts. The position of the patients during sleep was recorded with a body position sensor. With the help of the video camera system, audio and video recordings were made throughout the night; PSG recordings were scored according to the international criteria for sleep disorders using the TWin PSG analysis program (Grass Technologies Corporation, West Warwick, Rhode Island, United States) [17].

An airflow arrest of 10 seconds or more despite ongoing respiratory effort was scored as obstructive apnea, and a decrease in airflow of more than 30% with a 3% decrease in oxygen saturation for 10 seconds or more was scored as hypopnea. The average of apneas and hypopneas per hour of sleep time was calculated and defined as the apnea-hypopnea index (AHI). As part of the PSG analysis, the following values were recorded: total sleep duration, sleep stage duration, sleep latency, sleep stage latencies, number of awakenings lasting more than three minutes, sleep efficiency, AHI, oxygen desaturation index (ODI), minimum oxygen saturation, and time spent below 90% saturation during sleep. Patients were divided into three groups according to OSAS severity. Those with AHI:5-15 were grouped as mild OSAS, those with AHI:16-30 as moderate OSAS, and those with AHI:30 and above as severe OSAS, and the relationship with HbA1c was examined.

This study was conducted in accordance with the principles of the Declaration of Helsinki, and all patients provided written informed consent prior to enrollment. The authors disclose that artificial intelligence-assisted technologies, such as large language models (LLMs), chatbots, or image creators were not used in the production of the submitted manuscript. This study was approved by the Health Sciences University Süreyyapaşa Chest Diseases and Thoracic Surgery Training and Research Hospital Ethics Committee on July 6, 2023, with protocol number 116.2017.R-309.

Statistical analysis

Our data were analyzed via the IBM SPSS Statistics for Windows, Version 26, (Released 2019; IBM Corp., Armonk, New York, United States). Mean and standard deviation values were used in descriptive statistics. The distribution of the data was evaluated by the Shapiro-Wilk test. The chi-square test was used to compare categorical values. Independent samples t-test was used to analyze numerical data with normal distribution, and the Mann-Whitney U-test was used to analyze data without normal distribution. A p-value<0.05 was considered statistically significant.

## Results

In the study, patients were divided into two groups as diabetic and non-diabetic according to anamnesis and laboratory characteristics. Of the 50 diabetic patients, 70% were male and the mean age was 54.9 ± 9.3 years. Of the 42 non-diabetic patients determined as the control group, 57% were male and the mean age was 52.07 ± 9.1 years. The body mass indexes of diabetic and non-diabetic patients were found to be 34.5 ± 6.6 and 31.9 ± 7.1, respectively. No significant difference was observed between the two groups in terms of demographic data (Table 1).

**Table 1:** Demographic parameters in diabetic and non-diabetic patients *mean ± SD DM: diabetes mellitus; BMI: body mass index

Demographic parameter	Non-DM (n=42)	DM (n=50)	p-value
Age*	52.07 ± 9.1	54.9 ± 9.3	0.147
Gender, male (n (%))	24 (57.1%)	35 (70%)	0.200
BMI*	31.9 ± 7.1	34.5 ± 10.7	0.069

**Figure 1:**
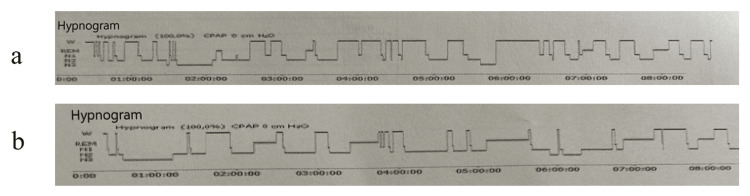
Hypnogram examples of the patients a. In a diabetic patient with obstructive sleep apnea syndrome (OSAS), the hypnogram showed frequent awakenings during sleep. The sleep architecture became fragmented and the duration of rapid eye movement (REM) sleep was short. b. In a non-diabetic patient with OSAS, the sleep architecture was nearly normal with very nice sleep and REM latency.

There was no significant difference between diabetic and non-diabetic patients in terms of total sleep duration, sleep efficiency, sleep latency, number of awakenings lasting more than three minutes during the night, and stage 1, 2, and 3 sleep duration, whereas REM latency was prolonged and REM sleep duration and percentage were significantly lower in diabetic patients (Figure 1) (Table 2).

**Table 2:** Sleep architecture findings in diabetic and non-diabetic patients *mean ± SD DM: diabetes mellitus; REM: rapid eye movement

Sleep architecture	Non-DM (n=42)	DM (n=50)	p-value
Total sleep time (minutes)*	437 ± 58.1	438.5 ± 51.4	0.897
Sleep latency (minutes)*	16.1 ± 12.7	12.6 ± 10.7	0.146
REM latency (minutes)*	154.5 ± 97.3	203.3 ± 121.5	0.039
Sleep efficiency (%)*	85.1 ± 10.9	83.7 ± 10.7	0.146
Number of awakenings lasting more than three minutes during the night*	4.6 ± 3.8	5.2 ± 4.3	0.435
Stage 1 sleep (%)*	3.6 ± 2.3	3.8 ± 2.9	0.772
Stage 2 sleep (%)*	60.3 ± 11.6	70.9 ± 50.6	0.188
Stage 3 sleep (%)*	14.3 ± 8.6	13.8 ± 13.6	0.840
REM stage (%)*	11.0 ± 8.1	8.3 ± 6.3	0.028

While no significant difference was observed between the nighttime minimum oxygen saturation values of diabetic and non-diabetic patients, AHI, ODI, and the time spent below 90% saturation during sleep, which indicate the severity of OSAS, were significantly higher in diabetic patients (Table 3).

**Table 3:** OSAS-related parameters in diabetic and non-diabetic patients *mean ± SD OSAS: obstructive sleep apnea syndrome; DM: diabetes mellitus

OSAS parameter	Non-DM (n=42)	DM (n=50)	p-value
Apnea-hypopnea index*	32.0 ± 20.8	46.1 ± 29.1	0.008
Oxygen desaturation index*	32.2 ± 23.1	45.2 ± 30.6	0.023
Minimum oxygen saturation value at night*	65.4 ± 13.9	63.1 ± 13.4	0.430
Time spent with oxygen saturation below 90% at night (minutes)*	16.5 ± 25.7	35.5 ± 33.6	0.003

**Figure 2:**
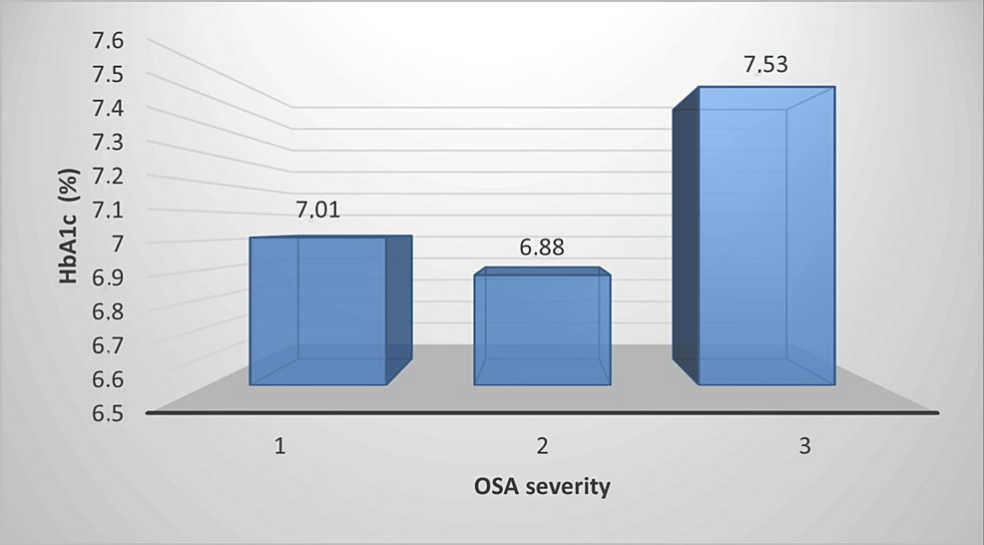
The relationship between OSAS severity and HbA1c in diabetic patients OSAS: obstructive sleep apnea syndrome; HbA1c: glycated hemoglobin

Diabetic patients were divided into three groups according to the severity of OSAS and HbA1c values were analyzed. The mean HbA1c of patients with mild OSAS (stage 1) was 7.0 ± 1.0 %, the mean HbA1c of patients with moderate OSAS (stage 2) was 6.9 ± 1.0 %, the mean HbA1c of patients with severe OSAS (stage 3) was 7.5 ± 1.6 %, and no correlation was found between the HbA1c parameter and the severity of OSAS, which we looked at in terms of diabetes control (Figure 2).

Diabetic patients were divided into two groups: HbA1c < 7 and HbA1c ≥ 7, and the relationship between diabetes control and sleep architecture was evaluated. No correlation was found between total sleep duration, sleep latency, sleep phase durations, sleep phase latencies, sleep efficiency, and the number of wakefulness lasting more than three minutes during the night (Table 4).

**Table 4:** The relationship between diabetes control and sleep architecture *mean ± SD HbA1c: glycated hemoglobin; REM: rapid eye movement

Sleep architecture	HbA1c < 7, (n=23)	HbA1c ≥ 7, (n=27)	p-value
Total sleep time (minutes)*	445.6 ± 60.1	432.4 ± 42.9	0.370
Sleep latency (minutes)*	12.5 ± 11.5	12.6 ± 10.1	0.985
REM latency (minutes)*	234.7 ± 139.9	176.5 ± 98.2	0.092
Sleep efficiency (%)*	82.8 ± 12.9	84.5 ± 8.6	0.596
Number of awakenings lasting more than 3 minutes during the night*	4.9 ± 5.2	5.5 ±3.3	0.670
Stage 1 sleep (%)*	3.2 ± 2.6	4.3 ±3.1	0.220
Stage 2 sleep (%)*	77.9 ± 73.9	64.9 ± 11	0.372
Stage 3 sleep (%)*	16.7 ± 17.4	11.3 ± 8.6	0.168
REM stage (%)*	8.9 ± 7.4	7.7 ± 5.1	0.480

## Discussion

In our study, REM sleep duration was significantly shorter and REM latency was prolonged in OSAS patients with diabetes. Furthermore, AHI, ODI, and time spent below 90% saturation in sleep were significantly higher in diabetic patients. The HbA1c parameter, which was examined in terms of diabetes control, was found to be higher in severe OSAS patients than in the other groups, although not statistically significant.

The presence of complaints such as accompanying neuropathic pain and nocturia due to hyperglycemia in diabetic patients may adversely affect sleep quality. Tare et al. found that short and long sleep duration was associated with increased Type 2 DM, while Barone et al. found that sleep deprivation posed a risk for glucose tolerance disorder [18,19]. Similarly, Pyykkönen et al. revealed that sleep duration of fewer than six hours or nine hours or more increased the risk of type 2 DM [20]. According to the National Sleep Epidemiology Study in Adult Population, which investigated sleep duration and insulin resistance, 13% of the population had difficulty falling asleep, 30% slept more than eight hours, and 11% slept less than six hours [21]. In our study, no significant difference was observed between the control group and diabetic patients in terms of sleep duration and sleep efficiency. These differences in the studies may be due to differences in the number of patients and the scales used. 

REM sleep is a physiological and repetitive behavioral state involving sustained neuronal activity corresponding to high brain energy requirements. In REM sleep, brain glucose utilization and cerebral blood flow increase [22]. It has been observed that insulin and glucagon concentrations decrease in REM sleep [23]. The brain, which makes up about 2% of the body, is completely dependent on glucose metabolism and uses about 50% of total body glucose [24], while it receives about 10% of blood glucose thanks to facilitated glucose transport that does not require insulin to cross the blood-brain barrier [25]. In many physiological aspects, REM sleep differs from other sleep stages, and brain activation is achieved with 30% more blood flow than in quiet wakefulness, which is associated with increased glucose consumption [26]. Furthermore, in a Brazilian study conducted by home PSG, 1074 participants were observed to have a shorter duration of REM sleep than those without diabetes. Even when potential confounding factors such as age, sex, body mass index, and AHI were excluded, shorter REM sleep duration was found in patients with diabetes [27]. Similarly, in our study, we found that REM latency was prolonged and REM duration was negatively affected in patients with DM. In patients with OSAS, diabetes-induced glucose metabolism disorder was found to negatively affect the REM phase, which is called paradoxical insomnia, and consumes the most glucose, which is vital in cognitive functions [28,29].

The Wisconsin sleep study was one of the first studies to detect the association between DM and OSAS. In this study, it was stated that the increase in AHI value and the increase in the frequency of diabetes were correlated with each other [30]. According to The Sleep Heart Health study, it was found that sleep disorders increased and more severe hypoxemia developed in individuals with DM [31]. Studies conducted to investigate the relationship between DM and sleep apnea have suggested that autonomic neuropathy due to DM may cause dysfunction of the respiratory center controlling the diaphragm and decreased upper airway tone. Reactive oxygen radicals resulting from intermittent hypoxia as a result of recurrent apneas throughout the night increased sympathetic activity, and systemic inflammation with neurohumoral and autonomic activation by stimulation of stress mechanisms due to changes in cerebral blood flow have been suggested to cause disturbances in glucose regulation and increased risk of developing DM [32].

There is an independent relationship between OSAS and type 2 DM, and DM suppresses basal ventilatory function. Hypoxia leads to decreased insulin sensitivity and increased cortisol and norepinephrine levels. In a meta-analysis published by Reutrakul et al., it was observed that the presence of DM in OSAS patients increased the severity of OSAS. Accompanying obesity is the main common risk factor for DM and OSAS [13]. In a descriptive article published by Muraki et al., it was reported that OSAS severity increased the risk of developing DM independently of obesity [12]. In our study, despite both groups having similar BMIs, the severity of OSAS and accompanying hypoxemia findings were significantly higher in diabetic patients. The time spent sleeping below 90% at night was higher in diabetic patients. In light of these data, we concluded that there is a bidirectional relationship between OSAS and DM as a result of a common multifactorial etiopathogenesis and that the presence of diabetes creates susceptibility for both the occurrence and progression of OSAS.

HbA1c level is a good indicator in determining diabetes regulation and the risk of developing diabetes-related complications. The HbA1c test is a glycosylated hemoglobin blood test that shows what the average blood sugar (plasma glucose) level has been in the last two to three months. In a prospective study involving 115 individuals with diabetes, an independent correlation was found between the AHI index during REM sleep time and increased HbA1c levels, whereas no such correlation was shown for non-REM AHI [33]. Similarly, a study from our country, Turkey, found that REM-related OSAS and severe OSAS were associated with diabetes, with differences in glucose values and increased HbA1c levels [34]. Meta-analyses revealed a strong association between OSAS severity and elevated HbA1c levels, even after controlling for multiple comorbidities [13]. In a study by Babu et al., significant decreases in postprandial blood glucose and serum HbA1c levels were found in patients with DM after three months of continuous positive airway pressure (CPAP) treatment [35]. A study by Herth et al. showed that CPAP treatment in patients with DM and OSAS was associated with a decrease in HbA1c, and there was also a correlation between CPAP duration and patient compliance with CPAP treatment [36]. In our study, although not statistically significant, the patients with the highest HbA1c values were the patients with the highest OSAS severity.

Limitations

This study has a relatively small sample size, potentially limiting the generalizability of findings to larger populations. The study relied on retrospective analysis of medical records, which may introduce limitations in data collection and interpretation. The study did not consider potential confounding variables such as physical activity levels, dietary habits, neck circumference, duration of diabetes, or other comorbidities, which could influence the relationship between diabetes and sleep architecture in patients with OSAS. Another weakness of our study is that the sample size was small and the effect of CPAP treatment on diabetes control could not be examined.

## Conclusions

OSAS and DM are two important diseases that are frequently observed in the community and negatively affect the quality of sleep. The co-existence of two diseases results in more severe outcomes and worse progress. Yet, sleep disorders are commonly underdiagnosed in patients with DM. Our study emphasizes the importance of awareness regarding sleep quality and sleep-disordered breathing in diabetic patients. Sleep is essential for both mental and physical well-being. In this regard, it is of utmost importance to examine diabetic patients for OSAS and to perform PSG in appropriate patients.
